# Scientific Items

**Published:** 1880-03-20

**Authors:** 


### SCIENTIFIC ITEMS.

**About Snakes.**—The question how snakes progress is answered by the books in a way satisfactory to many minds, but Mr. H. F. Hutchinson, who writes about them in a recent number of "Nature," takes some exceptions to the usual explanation. He seems to have been a careful observer of their habits, and concludes that terrestrial snakes move in one or the other of the following ways :

1. On smooth, plane surfaces, by means of their rib-legs; e. g., the boa.

2d. Through high grass, by a rapid, almost invisible, sinuous onward movement, as the hydrophidæ in water; e. g., the rat-snake.

3. Climbing trees, or ascending smooth surfaces by erecting their abdominal scales, or using them to produce a vacuum, as lizards do their foot-scales for ascending smooth surfaces; e. g., tree-snakes and cobras.

Mr. Hutchinson captured a snake nine inches long with a head less than half an inch broad, and presented it with a frog two inches long and one broad. The snake saluted the frog by seizing it by the nose. The animal made desperate attempts to shake it off, but in vain, and all the while the process of deglutition (?) was going on, or rather the snake was slowly but surely getting outside the frog. This was accomplished by a sort of vermicular process. The sharp little teeth were seen to advance slightly, and then the whole body wriggled up to a new hold on the frog. In this way it very gradually disappeared, the whole process lasting half an hour. The so-called snake-charming, Mr. Hutchinson is confident, is only clever *legerdemain*. He describes the operation of skin-shedding as follows : "The skin ready to be cast yields round the snake's mouth only, and remains adherent to the extremity of the tail. As the animal advances, the caudal extremity of the skin is inverted—that is pulled inward—and so the process goes on and is completed by the tail passing through the mouth of the skin; and thus the direction of the abandoned skin is directly opposite to the direction taken by the skin-casting snake—that is, if the mouth of the skin lies east, the snake went out to the west."—*Popular Science.*

**Voice in Fishes.**—Mr. S. E. Pool, in a late number of "Nature," gives an account of an interesting observation of his own in support of the claim that fishes possess a faculty of voice. He states that, when engaged in a survey of the "Disang River, in Eastern Assam" some six years ago, he had occasion to sound the depth of a pool. When seated in a small canoe and slowly nearing it, he suddenly became aware of the presence of a number of fish called "mahsir." They were evidently attracted by the canoe, and Mr. Pool surmised that they might possibly think it a huge dead fish. While watching their movements, he became aware of a peculiar "cluck" or percussive sound—frequently repeated, on all sides, and coming from below, but near by. This was soon traced to the mahsir, and one of them made distinct sounds which were answered by others. He further states that in some parts of Eastern Assam a large bivalve sings in concert with others.

**Submarine Telephoning.**—Mr. Charles Ward Raymond, C. E., describes, in Van Nostrand's Engineering Magazine, the result of some experiments with the telephone in submarine operations at depths not exceeding thirty feet.

One telephone (Phelps' Duplex) was placed in the diver's helmet, and fastened in such a position that, by simply turning his head, he could place his mouth or his ear to the instrument. The other telephone was placed on the screw which carries the air-pump and diver's helpers. Using Edison's carbon transmitter, with the addition of an induction coil and battery, the arrangement was perfectly successful. Conversation was carried on with the utmost facility; it was not necessary to give the diver any signal other than a simple "halloo!" It was also found that the diver could talk in the helmet without putting his mouth to the instrument and be heard plainly, and therefore he could continue his work and conversation at the same time. The battery, induction coil and transmitter were placed on a shelf on the diver's scow, and together occupied no more room than would a Webster's Unabridged Dictionary; the telephone in the helmet occupied but little room, and, of course, was not at all in the way.—*Druggists' Circular and Chem. Gazette.*

**Novel Theory About Diamonds.**—One of Dr. W. Fletcher's frogs escaped from his ranarium some time ago, and was found the other day behind a register at his office, starved to death and shrunk to half its former dimensions. The Dr. dissected it, and coming to its lungs found these organs clogged with thousands of black crystals which looked like coarse gunpowder. Under the microscope those crystals presented regular facets with smooth surfaces, presenting the same angles of crystallization as the diamond. On burning they gave off carbonic acid gas, and they are pure crystals of carbon, as the diamond is. According to the Indianapolis Herald, the doctor ingeniously theorizes that in the ages gone by, the huge reptiles of the antediluvian period, dying under circumstances similar to those under which the frog did, may have formed large crystals of carbon in their lungs which were afterwards transformed into the hard and lustrous diamond.—*Drug. Circ. and Chem. Gaz.*

**The King of Ploughs.**—A Chicago paper gives an account of the largest plough that was ever known to be made, recently turned out by an Illinois firm of agricultural machinery makers, for use on the St. Louis, Iron Mountain, and Southern Railway. It is calculated to cut a ditch thirty inches wide and two feet deep, and is worked by attaching it to a platform car of a construction train by means of timbers framed and extending out, so that the plough cuts its ditch a sufficient distance from the track. It cuts a furrow eight inches deep each time, requiring three of them to reach the proper depth, and it will make one mile of ditch two feet deep and three feet wide every four hours, thus doing the work of about one thousand men. The beam is made of swamp oak, and is eight inches by fourteen inches, the land side being made of bar iron eight inches wide and one and a half thick, which has to be forged expressly for the purpose. Its total weight is seventeen hundred pounds. The use of this plough will mark an era in all ditching work.—*Boston Journal of Chemistry.*

